# Spatio-temporal distribution and international context of bovine viral diarrhoea virus genetic diversity in France

**DOI:** 10.1186/s13567-024-01377-9

**Published:** 2024-10-03

**Authors:** Claire Lescoat, Delphine Perrotte, Séverine Barry, Élise Oden, Valentin Herbet, Gaël Beaunée, Marc Tabouret, Fabienne Benoit, Pierre-Hugues Pitel, Véronique Duquesne, Xavier Bailly, Julien Thézé, Guy Kouokam

**Affiliations:** 1 Université Clermont Auvergne, INRAE, VetAgro Sup, UMR EPIA, Saint-Genès-Champanelle, France; 2 Laboratoire LABÉO, Caen, France; 3 Ruminant Disease and Welfare Unit, National Reference Laboratory for BVD, ANSES, Ploufragan-Plouzané-Niort Laboratory, Niort, France; 4 Oniris, INRAE, UMR BIOEPAR, Nantes, France; 5 ANSES, Sophia Antipolis Laboratory, Sophia Antipolis, France; 6 French Federation of Animal Health Protection Groups (GDS France), Paris, France

**Keywords:** *Pestivirusbovis*, genotypes, 5’UTR, next-generation sequencing, phylogenetics, cattle, spatial structure, eradication plan

## Abstract

Bovine viral diarrhoea (BVD) is one of the most economically damaging livestock enzootic diseases in the world. BVD aetiological agents are three pestiviruses (BVDV-1, -2 and HoBi-like pestivirus), which exhibit high genetic diversity and complex transmission cycles. This considerably hampers the management of the disease, which is why eradication plans have been implemented in several countries. In France, a national plan has been in place since 2019. Our understanding of its impact on the distribution of BVDV genotypes is limited by the availability of French genetic data. Here, we conducted a molecular epidemiology study to refine our knowledge of BVDV genetic diversity in France, characterise its international relationships, and analyse national spatio-temporal genotypic distribution. We collated 1037 BVDV-positive samples throughout France between 2011 and 2023, with a greater sampling effort in two major cattle production areas. We developed a high-throughput sequencing protocol which we used to complete the 5’UTR genotyping of this collection. We show that two main BVDV-1 genotypes, 1e and 1b, account for 88% of genotyped sequences. We also identified seven other BVDV-1 genotypes occurring at low frequencies and three BVDV-2 samples (genotype 2c). Phylogenetic analyses indicate different worldwide distribution patterns between the two main BVDV-1 genotypes. Their relative frequencies present no major changes in France since the 1990s and few variations at the national scale. We also found some degree of local spatial structuring in western France. Overall, our results demonstrate the potential of large-scale sequence-based surveillance to monitor changes in the epidemiological situation of enzootic diseases.

**Supplementary Information:**

The online version contains supplementary material available at 10.1186/s13567-024-01377-9.

## Introduction

Bovine viral diarrhoea (BVD) is a major infectious disease of cattle that affects virtually all livestock producing countries worldwide [1–3]. The disease can lead to an important reduction in zootechnical performance, resulting in significant but heterogeneous economic impact among countries, estimated at up to 687 US dollar per animal in an infected herd [4]. Its huge socio-economic burden has led seventeen European countries to implement a control plan over the past thirty years [1, 4].

BVD can be transmitted horizontally from infected animals or fomites, resulting in a transient infection with various clinical outcomes in terms of symptoms and severity. They may include digestive, respiratory, reproductive symptoms and immunosuppression, but are typically mild [5]. BVD vertical transmission can occur and result in the birth of a persistently infected (PI) calf, depending on the stage of gestation at the time of infection [6]. PI animals propagate and sustain the disease and are essential to BVD endemicity [5].

BVD etiological agents are viruses from three species of the *Pestivirus* genus in the *Flaviviridae* family: BVD virus 1 (BVDV-1), BVDV-2 and HoBi-like pestivirus, currently recognized by the nomenclature of the International Committee on Taxonomy of Viruses [7] as *Pestivirus bovis*, *Pestivirus tauri* and *Pestivirus brazilense* respectively. Pestiviruses are single-stranded positive-sense enveloped RNA viruses of approximately 12.3 kb. Their genomes consist of one open reading frame coding for a polyprotein, flanked by two untranslated regions (UTR) [8]. Extensive molecular epidemiology studies mainly based on 5’UTR, N^pro^ and E2 partial regions have highlighted the subdivision of BVDV-1 into more than 24 genetic lineages (genotypes 1a to 1x) and the subdivision of BVDV-2 and HoBi-like pestivirus into 5 lineages each [2, 9–11].

In France, only three molecular epidemiology studies have been published, showing the predominance of BVDV-1 with two main genotypes (1e and 1b) [10, 12, 13], the sporadic presence of BVDV-2 [2, 10, 14] and the absence of HoBi-like pestivirus [10]. Our knowledge of the long-term trends of BVDV-1 genetic diversity in France and its spatial distribution is limited, although it could unveil different dynamics in the circulation of BVDV genotypes and help to understand BVD distribution. This information would be important as an eradication plan has been in place in France since August 2019 [15]. It relies on the identification and management of PI animals, either through the serological monitoring of herds by screening tank milk or blood samples, or by testing directly for viruses by qRT-PCR in ear notches from newborn calves (sample taken within twenty days of birth). In this context, monitoring BVDV genetic diversity is crucial to describe the genotypic distribution before and during the plan. It is also important to ensure that the surveillance and control methods in use, such as diagnostic tests and vaccines, remain appropriate considering the genotypes in circulation.

Here, we report BVDV 5'UTR genotyping data obtained from infected cattle in most regions of France from 2011 to 2023. We describe BVDV genetic diversity in France and its international relationships, and analyse the spatio-temporal genotypic distribution.

## Materials and methods

### Sample collection

**Figure 1:**
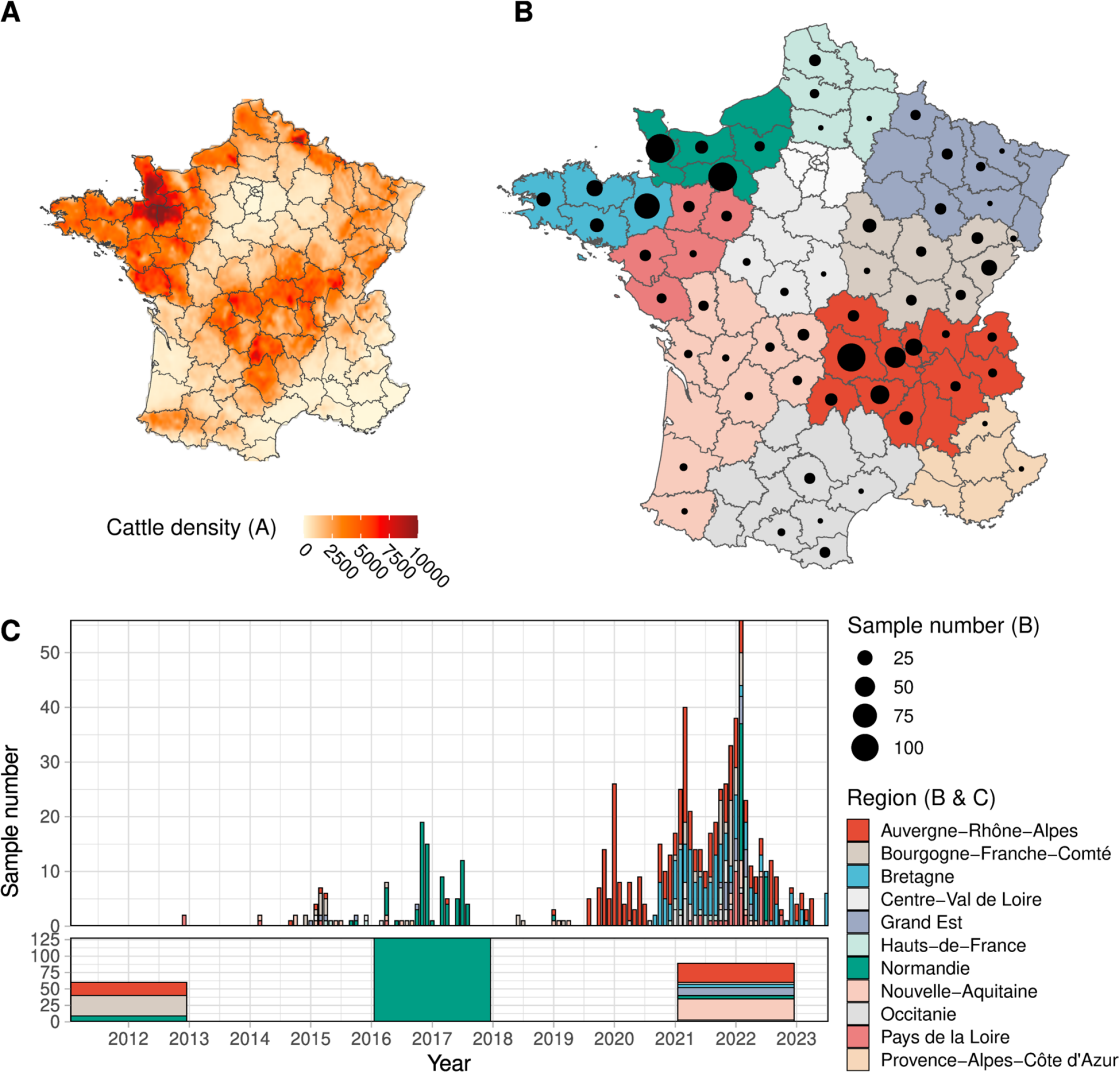
**Geographical and temporal distribution of Bovine viral diarrhoea virus samples in this study and cattle density.** **A** Map of France showing department administrative subdivision. The colour gradient indicates cattle density km^−2^ extracted from Robinson et al. [56]. **B** Spatial distribution of sample collection. Black circles represent the number of samples collected in each department. The map is coloured according to French administrative regions and was plotted using the R package sf [79]. **C** Temporal distribution of samples. The number of samples over time is presented in two panels, according to the level of precision of the metadata available for each sample: month and year of collection (top panel) or period only (bottom panel). Bar charts are coloured according to French administrative regions.

French BVDV-positive samples were obtained from four different sources (Figure 1 and Additional file 1). All samples have been diagnosed by regional veterinary analysis laboratories. Positivity was assessed mainly with PCR-based tests and, in few cases, with antigen ELISA-based tests. The first dataset (called dataset 1) represents BVDV-positive samples collected by the BVD national reference laboratory (NRL) between 2011 and 2022. Samples originated from most of the cattle-producing departments (2nd French administrative level, which corresponds to Nomenclature of Territorial Units for Statistics French—NUTS 3 level). It comprises samples collected as part of routine surveillance between 2011 and 2020 and targeted surveillance between 2021 and 2022 with ten random samples from different herds per department. Routine surveillance samples are of various origins in terms of tissues and animals. Targeted surveillance samples originate from ear notches collected as part of the eradication plan in France. The second dataset (called dataset 2) comprises BVDV-positive samples collected between 2016 and 2017 in the Normandie region (French NUTS 1 level). Samples were mostly blood and were isolated from both calves and adults, PI or not, in a variety of circumstances, including cattle introductions, sanitation plans, certifications or specific diagnostics commissioned by veterinarians. The two last datasets (called datasets 3 and 4) comprise BVDV-positive ear notches isolated from newborn calves as part of the French national BVD eradication plan. They originated from Auvergne-Rhône-Alpes (AuRA, dataset 3) and Bretagne (dataset 4) regions, collected during the periods August 2019—April 2023 and September 2020—April 2023, respectively. Additionally, BVDV-positive serum samples were collected in AuRA region in 2021. Each sample of our collection is associated with a location and date of sampling (Figure 1 and Additional file 1). For dataset 1, only the department of origin and the sampling year are available. For datasets 2, 3 and 4, we were able to retrieve the farm postcode and the sampling date for most of the samples. For dataset 4, the sampling date was approximated by the birth date of the calves.

### BVDV 5’UTR amplification and sequencing

All sequences obtained in this study are based on PCR amplification of 5’UTR using primers 326R and 324F [16]. RNA extraction step is detailed for each dataset in Additional file 2. Four distinct 5’UTR sequencing protocols were used. All 5’UTR amplicons obtained from samples in datasets 1 and 2 were sequenced using Sanger technology, as were 96 samples in dataset 3 (Additional file 2). 5’UTR sequences of the remaining samples in dataset 3 and all samples in dataset 4 were generated using a high-throughput sequencing protocol based on Illumina technology as described below.

Nucleic acids extracted from ear notches from datasets 3 and 4 (see Additional file 2 for details) were treated to remove DNAs using DNaseI (Invitrogen) at room temperature for 15 min, followed by inactivation with EDTA at 65 °C for 10 min. The resulting purified RNAs were reverse transcribed with SuperScript III reverse transcriptase (Invitrogen) using primer 326R [16] as described by the manufacturer (Invitrogen, 18,080–051) on a T100 PCR thermal cycler (BioRad). A high-throughput Illumina sequencing library preparation protocol was adapted from the 16S metagenomic sequencing protocol of the manufacturer (Illumina, 770–2018-009-B). In brief, it consists of using primers composed of a locus-specific sequence, i.e. primers 326R and 324F [16], and Illumina adapters added to primer sequence extremities (Table 1) for direct sequencing of short amplicons on an Illumina machine without DNA tagmentation. PCR amplification was performed using the modified primers and Q5 polymerase enzyme (New England BioLabs). The reaction was carried out using the temperature cycle described in Additional file 3. Samples were purified using Macherey–Nagel NucleoMag NGS Clean-up and Size select with a beads ratio of 3:2 and normalised to 0.2 ng/µL prior to the next step. Illumina dual-index barcodes were added for multiplexing amplicons with the Nextera^™^ XT DNA Index Kit using a limited cycle PCR (see Additional file 3). Quality and average size of the libraries were checked using an Agilent 2100 Bioanalyzer High Sensitivity DNA chips following the manufacturer’s recommendations. Each library was purified as described above, quantified in Qubit with a Qubit dsDNA HS assay kit (ThermoFisher), normalised and then mixed to obtain a pool of 96 libraries at 1 nM. BVDV libraries were diluted to 70 pM and pooled with 15–20% of PhiX (Illumina) 100 pM or a 70 pM tick mitochondrial DNA library (data not published) in order to increase nucleotide diversity. These additional libraries had a higher genetic diversity than 5’UTR amplicons and help to provide a more balanced signal for the instrument. The latter library was obtained using the same protocol as presented here. Three sequencing runs of 96 samples each were performed on an Illumina iSeq100 benchtop sequencer using 20 µL of the pooled libraries (paired-end runs and 151 cycles).

**Table 1:** **5’UTR primers adapted from Vilček et al.** [16] **(in bold) with Illumina adapter sequences.**

Primer name	Sequence (5’—3’)
324F-iseq100	TCGTCGGCAGCGTCAGATGTGTATAAGAGACAG-**ATGCCCWTAGTAGGACTAGCA**
326R-iseq100	GTCTCGTGGGCTCGGAGATGTGTATAAGAGACAG-**TCAACTCCATGTGCCATGTAC**

### Assembly from high-throughput sequencing

Raw read quality was assessed with FastQC v0.12.1 [17] and quality reports were aggregated using multiQC v1.12 [18]. Reads shorter than 10 nucleotides were discarded. Primers, adapter sequences and flanking N bases were trimmed using cutadapt v4.2 [19]. Quality trimming was carried out using Trim-Galore v0.6.7 [20] with a quality threshold of 30. All samples were aligned to the 5’UTR of NADL reference strain (GenBank accession number M31182). The reference sequence was indexed using Burrows–Wheeler Aligner (BWA) v0.7.17 and samtools v1.16.1 [21, 22]. Reads were mapped to the reference sequence with the BWA-MEM algorithm. A Bayesian approach to variant calling was performed using FreeBayes with ploidy set at 1 and a min-coverage of 10 [23]. VCF files were filtered using VcfFilter v1.0.9 to remove any site with estimated QUAL inferior to 20. Coverage was assessed from bam files using bedtools v2.30.0 genomecov -bga option [24]. Consensus sequences were produced with bcftools v1.16 and positions with depth coverage less than 10X were masked.

Analysis was conducted using R v4.3.0 [25] and Rstudio [26]. This pipeline is implemented in Snakemake v7.24.0 [27]. The following Snakemake wrappers were included: fastqc (v1.3.2), multiqc (v1.3.2), cutadapt/pe (v1.3.2), bwa/index (v1.19.1) bwa/mem (v1.3.2) samtools/index (v1.3.2) and freebayes (v1.3.2).

### Public BVDV-1 sequence collection

We collated all *Pestivirus bovis* (BVDV-1) genetic data publicly available from the National Center for Biotechnology Information (NCBI) on 24^th^ August 2023. Using in-house scripts based on Python module BioPython [28], sequences were searched using the taxon identifier of *Pestivirus bovis* (id: 2170080) and all his descendants except BVDV-2a (id taxa: 1855269) and BVDV-2b (id taxa: 2590438) as determined by the NCBITaxa function of the ete3 module [29]. A total of 21,989 BVDV-1 sequences were retrieved, covering mainly the 5’UTR (16 933 with “5’UTR” in GenBank description). Sequences less than 100 nucleotides long were removed (*n* = 83), resulting in a dataset of 21 906 public sequences.

We selected a subset of 113 sequences, of which 104 represented genotypes 1a to 1x  for BVDV-1 and 9 represented genotypes 2a to 2e for BVDV-2 (Additional file 4). Some recently assigned genotypes have a conflicting letter code and were distinguished by the country of publication. These are genotypes 1l_Turkey [30] and 1l_France [12]; 1r_Italy [31] and 1r_Turkey [32]; and 1v_Turkey [33] and 1v_China [34].

Another subset of 214 publicly available sequences was selected comprising only sequences isolated in France from cattle. It included 6 sequences from Vilček et al. [13] corresponding to 5 isolates, 61 sequences from Jackova et al. [12] corresponding to 47 isolates, 145 sequences from Rivas et al. [10], as well as two others sequences: FJ493479 and JQ994205. Sequences from different genomic regions with the same isolate information were concatenated, resulting in a dataset of 199 sequences.

### BVDV-1 public sequence genotype assignment

An exhaustive genotype assignment of publicly available BVDV-1 sequences was performed. Taxonomic assignment was carried out in three steps. Briefly, the 21 906 public sequences were clustered using the CD-HIT program [35] with a similarity threshold of 99%. A total of 5636 clusters were defined and the representative sequence of each cluster was retrieved. Batches of 100 representative sequences were aligned to the reference sequences of BVDV-1 genotypes (Additional file 4) using MAFFT version 7.490 [36] with add and localpair parameters. Maximum likelihood (ML) phylogenetic trees were inferred for each batch using IQ-TREE v.2.2.0 [37] with the nucleotide substitution model GTR + F + R7 and 1000 ultrafast boostrap (UFBoot) [38] (minimum correlation coefficient for UFBoot convergence: 0.95). The genotype of each representative sequence was assigned based on its inclusion in genotype clades of reference sequences using ML trees and the mrca function in the R ape package [39]. Sequences not included in genotype clades of reference sequences were assigned to the genotype of the closest reference sequence according to cophenetic distance matrix (cophenetic.phylo function in ape R package [39]) and branch statistical support (UFBoot). Genotypes were assigned to 5322 representative sequences, and by extension, to corresponding clusters, resulting in a total of 21 261 genotyped sequences. To reduce redundancy per genotype, sequences were finally clustered by country (or “unknown” in case of missing values) with a similarity threshold of 99.5% using CD-HIT program.

### Phylogenetic analyses

Four multiple sequence alignments (MSA) were generated. One alignment included reference sequences, previously published French sequences and sequences of this study. The other three MSA were created specifically for genotypes 1e, 1b and 1d, and included publicly available sequences and sequences of this study assigned to each genotype. Sequences were aligned using MAFFT version 7.490 [36]. MSA were manually curated using Aliview v1.28 [40]. IQ-TREE v.2.2.0 [37, 41] was used to infer maximum likelihood phylogenies based on each of the four alignment with the best-fitting nucleotide substitution model and parameters estimated by ModelFinder embedded in IQ-TREE. Generalised Time Reversible (GTR) substitution model was selected for all datasets with an empirical state frequency (F) and a free rate heterogeneity (R) with different categories depending on the alignment (GTR + F + Rx). Ultrafast bootstrap procedure [38] and Shimodaira–Hasegawa approximate likelihood ratio test (SH-aLRT) [42] were used to assess statistical branch supports for the first dataset. Phylogenetic trees were visualised using R package ggtree [43]. A root-to-tip analysis using TempEst [44] was performed to measure the molecular clock signal contained in the 5’UTR sequence data. The linear regression between sample collection dates and root-to-tip genetic distances showed very weak correlation (R^2^ < 0.0005) indicating that a molecular clock approach based on 5’UTR sequences would not be robust.

### Spatial and temporal statistical associations

Fisher exact tests (FET) [45] were used to test the independence between sampling periods or geographical areas and genotype relative frequencies. For large contingency tables (i.e., periods vs. genotype relative frequencies table), *p*-value was computed by Monte-Carlo simulation using fisher.test function of stats R package. When a significant association was identified (*p*-value ≤ 0.05), a post-hoc test was performed to determine the significance of the pairwise differences using fisher.multcomp function from RVAideMemoire R package [46]. *P*-values were adjusted using Benjamini–Hochberg correction. Spatial structure for samples with known farm postcode was assessed using Mantel correlograms, which estimate the correlation between genetic and geographic distances. Correlations were based on patristic distances between French samples with known farm postcode in the phylogenetic tree (Additional file 6) and geographic distances between centroids of corresponding postcodes. Mantel correlograms were computed using the mantel.correlog function from the vegan package, with Benjamini–Hochberg correction (options progressive = T and cutoff = F) [47]. Distance classes were determined using Sturge’s rule.

## Results

### Sample collection

The study included 1037 BVDV-positive samples collected in France between 2011 and April 2023 (Additional file 1). They originated from different sources (referred to as datasets 1–4, see Materials and Methods) associated with different sampling schemes. These schemes were either opportunistic (datasets 1 and 2) or random in specific geographical areas (datasets 1, 3 and 4). Most French cattle-producing regions are represented (Figures 1A, B) and we were able to collect more samples from three French regions (AuRA, Bretagne and Normandie), which are part of two main cattle production areas in France (Figures 1A, B). Due to the diversity of our sampling sources, the spatial distribution of samples is not homogeneous over time (Figure 1C). This is particularly the case for samples isolated in Normandie which were mainly collected in 2016 and 2017. The number of samples also varies greatly from one year to another, with most samples collected from 2020 onward as part of the eradication plan (Figure 1C).

### 5’UTR high-throughput sequencing

A high-throughput 5’UTR sequencing protocol was applied on 282 samples isolated from ear notches, included in datasets 3 and 4 (Additional file 1). These samples have diverse viral loads, with qPCR cycle threshold values (Ct) ranging from 18.28 to 34.89. The number of reads mapped per sample ranged from 3 to 438,597 (median of 22 548.5). The mean depth coverage varied from 1.3 to 213 795 (median of 9368.7). Most of the samples (270 out of 282, 96%) covered > 80% of the reference sequence. The remaining 12 samples had low coverage (< 65%), including 7 samples with no coverage, and were therefore not included in further analyses. These samples were sequenced in the same sequencing run (run 3) which showed a slight decrease in the number of mapped reads per sample compared to the other sequencing runs (Additional file 5A) and a much lower percentage of mapped reads (Additional file 5B). The lower efficiency of this third run was likely due to a high concentration of primer dimer. This is consistent with the mean read lengths of the three runs, which are 141 nucleotides (nt) (run 1), 142 nt (run 2) and 79 nt (run 3). Overall, a correlation is observed between the percentage of mapped reads and initial viral loads (Additional file 5B).

### BVDV genetic diversity in France

Of the 1,025 BVD-positive samples successfully sequenced in this study, only three samples were identified as BVDV-2 (genotype 2c), with all the others belonging to BVDV-1 (Additional file 1). This is consistent with previous studies highlighting the rarity and absence of BVDV-2 and Hobi-like pestivirus in France, respectively [10, 12–14]. Consequently, we focused exclusively on BVDV-1 in the subsequent analyses. The 5’UTR sequences of the 1,022 successfully sequenced BVDV-1 samples were aligned with 199 previously published French sequences and 104 reference sequences defining BVDV-1 genotypes (including 9 from France representing genotypes 1x and 1l_France). A maximum likelihood phylogenetic tree was inferred from this alignment of 1316 sequences (Additional file 6). The tree showed that the French sequences of this study were mostly divided into five distinct genotypes, including 580 sequences in 1e (57%), 327 in 1b (33%), 71 in 1d (7%), 13 in 1a (1.3%), 12 in 1l_France (1.2%). The 19 remaining sequences fell into 1k, 1i, 1r, 1s clades and into undetermined intermediate genotypes (see Additional files 1 and 6). Previously published French sequences were distributed throughout the tree, suggesting that we did not uncover any major unreported BVDV-1 genetic diversity in France. Only genotypes 1k and 1i have not previously been reported in France, but they account only for four sequences. Three samples (BVDV1Fr0209, BVDV1Fr0262 and BVDV1Fr0916, see Additional file 1, highlighted by an asterisk on Additional file 6) were identical or almost identical to vaccine strains KE-9 (genotype 1b; Bovela, Boehringer Ingelheim) or Oregon C24 (genotype 1a; MUCOSIFFA, CEVA Santé Animale). An epidemiological survey confirmed that two out of the three samples were related to vaccinated animals (NRL for BVD, personal communication), consistent with the detection of these vaccine strains elsewhere [48]. No information was available for the last sample. The phylogeny shows an overall lack of support and a low phylogenetic resolution with numerous polytomies, especially for samples with close sampling dates and location in 1e clade.

### French genetic diversity of BVDV-1 in a global context

**Figure 2:**
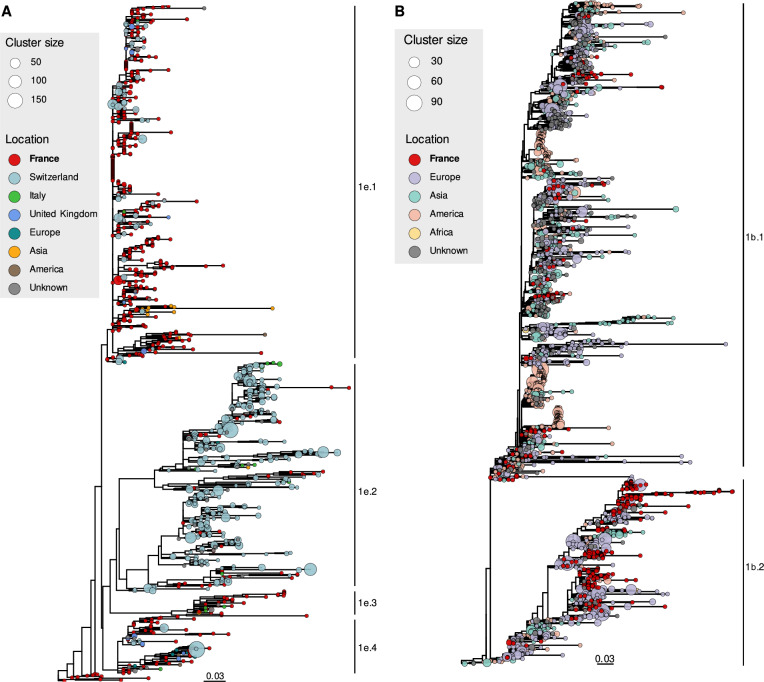
**Phylogenetic relationships between French and international sequences of BVDV-1 genotypes 1e and 1b.** Maximum likelihood phylogenetic trees inferred from French and international sequences of genotypes (**A**) 1e and (**B**) 1b. Tips are coloured according to the sample continent or country of origin and sizes represent the number of closely related publicly available sequences (99.5% identity) from the same location. European countries with less than ten sequences were grouped under the location “Europe” in 1e phylogeny.

The international context of the French BVDV-1 genetic diversity was investigated for the main genotypes identified in France (i.e. 1e, 1b, 1d, 1a and 1l; Figure 2, Additional files 7 and 8). We genotyped all publicly available BVDV-1 sequences on the NCBI database (*n* successfully genotyped = 21,261) and explored the most common location of samplings. Genotypes 1a and 1b were present in almost all cattle-producing countries, while genotype 1d has been mainly found in Europe (Additional files 7A and B). Genotype 1e has historically been sampled mainly in Western Europe, and genotype 1l_France has been identified almost exclusively in France (Additional file 7A and B).

The phylogenetic tree showing relationships between French and international sequences (Figure 2A) revealed that genotype 1e can be divided into four monophyletic groups called clades 1e.1 to 1e.4 for clarity. Most of the French genetic diversity of genotype 1e fell into clade 1e.1 with 488 sequences (75.3% of total 1e French sequences), while clades 1e.2, 1e.3 and 1e.4 comprised 47 (7.3%), 35 (5.4%) and 67 (10.3%) French sequences respectively. Clade 1e.1 was characterized by an overall low phylogenetic resolution compared to the three other 1e clades, with in particular an important polytomy at internal nodes. French sequences predominate (54.2% of total sequences in clade 1e.1) and are distributed between internal and external nodes. Clade 1e.2 included mostly sequences sampled in Switzerland between 2008 and 2012 as part of the Swiss national eradication plan [49]. French sequences from several regions were scattered throughout the clade, mainly at external nodes. Clade 1e.3, which corresponded to genotype 1x  in Rivas et al. [10], and clade 1e.4 contained mostly French sequences distributed at external nodes. Overall, genotype 1e phylogeny showed a marked difference between clade 1e.1 and the rest of the tree, suggesting a primary contribution of this clade to BVD disease in France.

The phylogeny of genotype 1b (Figure 2B) including French and international sequences can be divided into two monophyletic groups called clades 1b.1 and 1b.2 for clarity, with most of the French sequences falling into clade 1b.2 (241 sequences, 58.6% of total 1b French sequences). Clade 1b.1 contained sequences from most of the continents, while clade 1b.2 sequences were comparatively more sampled in Europe. French sequences were distributed throughout the tree into dozens of monophyletic groups at external nodes. International sequences fell into both internal and external nodes. The tree showed a clear mix of French and international sequences, suggesting a high rate of international migration, including several introductions into France that have contributed greatly to BVD disease.

The phylogenetic tree corresponding to genotype 1d (Additional file 8) showed three monophyletic groups, called 1d.1, 1d.2 and 1d.3 for clarity. Clade 1d.2 includes most of the French sequences (70 sequences, 90.9% of total 1d French sequences), which clustered in one subclade that was not particularly closely related to international sequences. The rest of the French sequences are scattered throughout the tree. Given the small number of 1a and 1l_France sequences identified, no phylogenetic trees were reconstructed with international sequences for these genotypes.

### Spatial and temporal distribution of BVDV-1 genotypes in France

**Figure 3:**
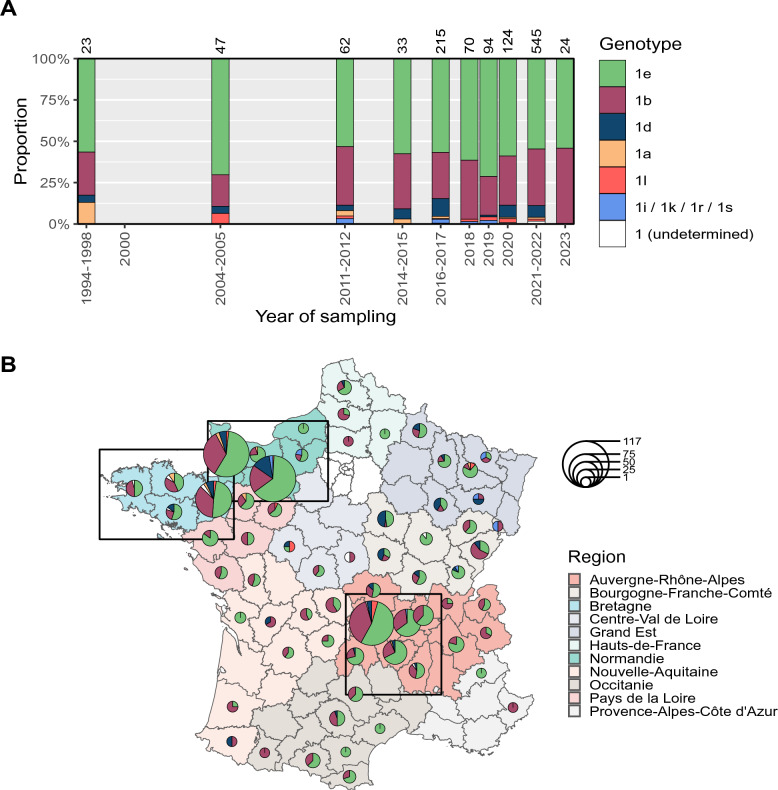
**Temporal and spatial distribution of BVDV-1 genotypes in France.** **A** Proportion of genotypes over time in France. Genotyping data include current and previous studies (1994–1998: Vilček et al., 2001 [13], 2004–2005: Jackova et al., 2008 [12]; 2018–2020: Rivas et al., 2022 [10]). Rare genotypes (1r, 1s, 1k, 1i) were grouped. Numbers at the top of bar charts indicate the total number of samples for each period. **B** Proportion of each genotype per French department over the 2011–2023 period. Genotyping data includes current study and those from Rivas et al. [10]. Pie charts denote genotype proportions per department and sizes are proportional to the number of samples. Black boxes show the location of the three focused regions: Bretagne, Normandie and Auvergne-Rhône-Alpes.

We investigated whether the distribution of BVDV-1 genotypes was structured in space and time. We collated all genotyping data available for France, including the present work and previous studies [10, 12, 13] ranging from 1994 to 2023 (Figure 3A), and examined the potential change in genotype relative frequency over this time interval. Periods were defined on the basis of the least precise available temporal information. A Fisher exact test (FET) comparing the proportion of genotypes (that is, 1e, 1b and all other genotypes grouped as “others”) including all periods was significant (*p*-value = 0.036), but a post-hoc analysis revealed no significant pairwise comparisons between periods. This analysis suggests that there has been no significant shift in the proportion of BVDV-1 genotypes in France since the 1990s, with a higher degree of confidence for the period 2018–2023 due to a more extensive national sampling (Figure 1C and Rivas et al. [10]).

The proportion of BVDV-1 genotypes by department in France is shown on Figure 3B. Of the previous French genotyping data, only that of Rivas et al. [10] was included along with the present data, as it contains information on the department of sampling and is included in the time-frame of this study (2011–2023). Genotypes 1e and 1b were the main genotypes in most of the well-sampled regions (*n* ≥ 30, mean relative frequencies of 1e and 1b were 57% ± 6% and 32% ± 8%, respectively). We compared differences in the relative frequencies of genotypes between regions using a FET. Test was significant (*p*-value = 0.0005), and post-hoc tests indicated also 27 significant pairwise comparisons (Additional file 9A), more than half of which (17 out of 27) included Grand-Est or Bourgogne-Franche-Comté (BCF) regions, both located in the North-East part of France. The majority of significant comparisons involved “others” genotypes and 1e or 1b, suggesting that the ratio of the main genotype proportions was stable across well-sampled regions of France. The same test was applied within regions for 24 departments with at least ten samples localized in seven regions: Normandie, Bretagne, AuRA, Occitanie, Grand Est, Pays de la Loire and BCF. FET was significant only for BCF region (*p*-value = 0.0005). Post-hoc tests showed significant differences between three departments: Yonne (code: 89), Doubs (25) and Jura (39) (Additional file 9B). This suggests few variations in the relative frequency of genotypes on a national scale, with some regional specificities such as in the North-East of France. Nevertheless, we cannot exclude the existence of significant divergent trends that are not captured by our dataset.

### Spatial structure of BVDV-1 genetic diversity

**Figure 4:**
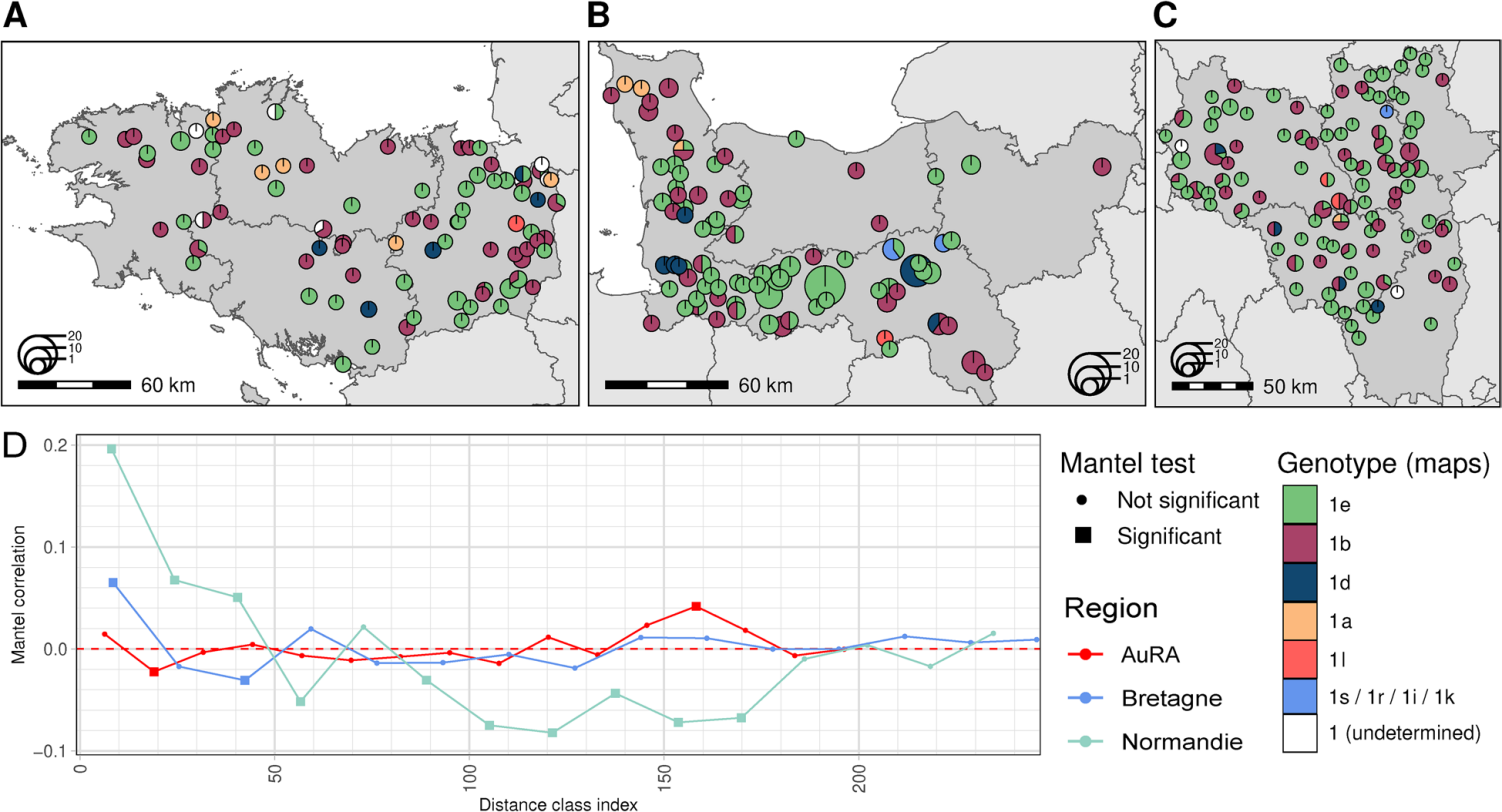
**Spatial distribution and local structure of BVDV-1 genotypes in three focused French regions.** Proportions of BVDV-1 genotypes at local scale in (**A**) Bretagne, (**B**) Normandie (**B**) and (**C**) Auvergne-Rhône-Alpes. Pie charts denote genotype proportions per farm postal code and sizes are proportional to the number of samples. A small amount of random variation was added to each pie chart location to ensure anonymity. Grey lines outline department borders. **D** Mantel correlograms measuring correlations between phylogenetic and geographical distances for the three focused regions. Each dot is positioned in the middle of a distance class. Statistically significant correlations are indicated by a square.

The geographical structuring of BVDV-1 genetic diversity was investigated at a regional scale for three well-sampled regions of the study (Bretagne, Normandie and AuRA, i.e. datasets 2, 3 and 4, Additional file 1). Only samples with known farm postcodes were included in the subsequent analyses. Maps of genotype proportions by farm postcode for these regions (Figures 4A, 4B and 4C) did not show strong geographical structuring of genotypes at the scale of a region or a department, although local clusters of similar genotypes can be observed, notably for Normandie. We therefore used Mantel correlograms to explore population structure relationship to space (Figure 4D). The correlations were very weak overall, and the correlograms shapes vary according to the region under consideration. Pairwise distances were significantly correlated for Normandie region (Figures 4B, D), positively in the first distance classes (up to 50 km) and negatively for longer distances (between 50 and 180 km). This describes an increasing genetic dissimilarity between pairs of samples as the geographical distance increases. Significant correlations were also observed for Bretagne region (Figures 4A, D) for smaller geographical scales: positively first (0–17 km), then negatively (34–51 km). AuRA region (Figures 4C, D) correlogram showed significant correlations for two distances classes, 12–25 km and 151–164 km, but cannot be interpreted. The lower magnitude of autocorrelation observed in AuRA region could be due to the longer time frame, that is four years of observations compared to two (Normandie) or three (Bretagne) years. To rule out this hypothesis, Bretagne and AuRA datasets were truncated to two years (2020–2022 for Bretagne and 2019–2021 for AuRA), but results showed similar trends compared to complete dataset results (Additional file 10). These results suggest that spatial structuring of BVDV-1 genetic diversity exist at local scales but vary depending on the region.

## Discussion

### Efficient high-throughput sequencing protocol for 5'UTR sequencing

In this study, we report a large number of BVDV 5'UTR genetic data (> 1000 samples) obtained from infected cattle in France between 2011 and 2023. This dataset includes sequences generated with a high-throughput sequencing protocol targeting *Pestivirus* 5’UTR that we have developed. This protocol is fast, reliable and can work on an Illumina lab-benchtop sequencing machine such as the iSeq100. With this high-throughput sequencing protocol, we successfully sequenced 270 out of 282 samples covering the complete targeted loci length. The small size of the 5'UTR and the efficiency of the Vilček et al. primers [16], make it possible to use this protocol to obtain robust sequencing when using low amounts of poor-quality samples, such as ear notches stored at −20 °C for several months. Given the mean coverage depth obtained here, the number of samples that can be multiplexed in one sequencing run is potentially high (at least 96 samples). These are two advantages compared to Sanger sequencing [50], which is the commonly used method to sequence 5’UTR BVDV amplicons [10, 48, 51]. Moreover, it requires only basic laboratory equipment to prepare the libraries, and is relatively inexpensive as no DNA tagmentation step during the sequencing library preparation is required. Finally, the conserved domains in the 5'UTR and the universality of the primers used enable monitoring of genetic diversity without a priori on virus species or genotype as it is possible to detect several pestiviruses [16]. Although the cost per sample is almost twice that of Sanger, the high sequencing success rate and the high level of multiplexing make our sequencing approach cost-competitive. This could facilitate future large-scale diversity studies of *Pestivirus*-related diseases.

### Circulation of two main genotypes of BVDV-1 in France

Several *Pestivirus* species capable of infecting cattle are of interest for health management: *P. bovis* (BVDV-1), *P. tauri* (BVDV-2), *P. brazilense* (HoBi-like pestivirus), and *P. ovis* (Border Disease Virus) [52]. However, publication on *Pestivirus* genetic diversity in France is scarce [10, 12, 13]. In this study, we collected sequences of BVDV-positive samples with a fairly representative sampling of cattle distribution in France. In particular, we acquired samples from major cattle production areas: Normandie, Auvergne-Rhône-Alpes (AuRA) and Bretagne. To date, this type of extensive sampling for molecular typing has only been carried out and published in Switzerland [51] and the United-Kingdom [53]. Despite its low phylogenetic resolution [9, 54], 5’UTR has been used in the vast majority of previous taxonomic studies, which facilitate comparisons. Taxonomic assignment of the samples of this study based on partial 5’UTR sequences shows the presence of two major BVDV-1 genotypes (1e and 1b), three minor genotypes (1d, 1a and 1l_France, subsequently termed 1l) and several marginal genotypes (1s, 1r, 1i and 1k) in France. This genetic diversity is consistent with previous observations [10, 12, 13], with the exception of 1k (Bourgogne-Franche-Comté, 2011/2012) and 1i (Normandie, 2016/2017) which have not been reported to date in France. Genotypes 1r (Normandie, 2016/2017) and 1s (AuRA, 2019) were previously observed in the East of France (in 2018 and 2019 respectively [10]). Of note, clade 1x defined in Rivas et al. [10] based on 5’UTR sequences clustered here among 1e sequences. The weak branch supports observed in the phylogenetic tree, combined with the absence of clear criteria for clade delimitation, suggest that genome-based tree reconstructions would be needed to clarify the status of this clade. Only BVDV-1 and -2 (genotype 2c) were identified here. We did not find isolates belonging to the *Pestivirus ovis* species, although most diagnostic tests can detect both BVDV and BDV [55]. This could partially be explained by our sampling scheme, which intended to characterize the broad genetic structure of circulating BVDV. Indeed, the detection of *P. ovis* would have required a more specific sampling design with a focus on mixed cattle-sheep farming systems; and higher sheep densities are in general found in regions that were less sampled in our study, such as the South of France [52, 56].

### Distinct international context between main French BVDV-1 genotypes

An exhaustive taxonomic assignment of publicly available 5’UTR sequences, combined with phylogenetic tree reconstructions, was performed to provide a better understanding of international virus flows. Genotype assignment analysis was complex due to the short length of studied sequences (dataset is composed of 75% 5’UTR sequences), errors in sequences and low phylogenetic signal of 5’UTR marker. These limits prevented us from constructing a well-supported phylogenetic tree directly from all publicly available sequences and using standard genetic clustering algorithm to confirm genotype assignment. Nevertheless, our results were overall consistent with the literature review of BVDV-1 genetic diversity by Yeşilbağ and collaborators [2]. Some inconsistencies could be due to differences with genotype assignment method in previous papers as reviewed in Yeşilbağ et al. [2], genetic diversity described but not publicly available such as 1l sample from Italy [57] or missing country annotation in GenBank (e.g., only nine publicly available sequences are annotated with Belgium as country of collection, whereas twenty are listed in paper by Yeşilbağ et al. [2]).

Each country seems to have its own genotype combination. France is characterized by a low number of genotypes and a predominance of the genotype 1e. This genotype has mainly been sampled in France and neighbouring countries, such as Switzerland [49] or Italy [57, 58]. Most of the French sequences group into one main clade (1e.1), that may have contributed primary to BVD infections in France. The potential contribution of under-sampled geographical areas to this clade cannot be ruled out due to the sampling bias in the publicly available dataset. Genotype 1b has been sampled in almost all cattle-producing countries. French sequences were distributed into different lineages within genotype 1b phylogeny, and grouped with sequences from all continents. This suggests several introduction events into France which gave rise to numerous transmission chains that contributed greatly to BVD disease. These results highlight different international context between genotypes 1e and 1b. The presence of rare genotypes (1s, 1k, 1r and 1i) in France is probably due to migrations from under-sampled French region or other countries. However, it is not clear whether their presence is due to the cryptic maintenance of transmission chains or to one-off events.

Migration rates between countries and their temporal dynamics are important to reconstruct transmission routes and understand the impact of cattle movements from abroad to BVD circulation dynamics in France. Due to the low phylogenetic resolution of 5’UTR marker, it was not possible to further explore the temporal dynamics of this clade in the present study. BVDV-1 could have been introduced into France on several occasions through different routes: mainly live cattle imports, closer contact between herds at borders, or import of contaminated semen. Changes in observed genetic diversity through the migration of one or more genotype could be expected as a result of large cattle movement from another geographical area with a different genetic diversity profile [59, 60]. However, the small number of live cattle imported into France [61] and the low probability of importing BVD-positive cattle put the current impact of this route into perspective. Indeed, only a handful of animals imported during the Switzerland eradication plan (2008–2012) was BVD-positive [62], even if this observation is to be considered with caution as “Trojan cows” (pregnant dams carrying a PI foetus) number can be underestimated [63, 64]. Close contacts at borders are hard to quantify without further data, but could be low as only Belgium and Luxembourg share a common border with France without a natural barrier such as the Rhine for Germany or mountains (Alps or Pyrenees) for Italy, Switzerland and Spain. Common grazing areas can be found in both mountain ranges but the extent of infection made possible by transhumance remains to be determined. German sequences of genotype 1d [64] did not particularly cluster with French sequences (Additional file 8). On the contrary, French and Swiss sequences are found relatively interspersed in clades 1e.1 and 1e.2 (Figure 2A), which supports the hypothesis of small-scale but frequent contacts between herds. A similar pattern could be expected with Italy due to the high proportion of 1e in North Italy [57, 58], but less sequences were retrieved or successfully genotyped. Finally, infections through semen are limited but possible [65], and imports could have impacted virus entry rates more likely decades ago as there were historical biosecurity recommendations but no mandatory controls on frozen semen [66]. Effective migration rate (i.e. migration events resulting in at least one local infection) is assumed to be much lower than real migration rate as PI presence in a herd tends to result in its seroconversion, providing herd immunity. This should limit the risk of introduction of another BVDV strain and facilitate the local maintenance of genotypes for years [67]. In addition, cross-reactivity have been demonstrated among BVDV-1 strains [68], and to a lesser extent between BVDV species [69]. Thus, effective migration rate into France should be low due to herd immunity for BVDV-1 genotypes and presumably higher for BVDV-2 and Hobi-like pestivirus. It is rather the lower international prevalence that may explain the rarity of these pestiviruses in France. However, phylogeny structure may reflect biases in available data and miss nodes related to key migration events. Publicly available sequences are biased geographically (some countries or areas are under-sampled), temporally (most data come from studies conducted over limited periods of time, as opposed to regular monitoring), in terms of local context (e.g. eradication plan or mitigation measures, such as vaccination) and sampling scheme. As a result, the clustering of French and international samples cannot be interpreted as direct evidence of migration as geographical, temporal and potentially genetic structures are confounded in the data, and the temporal aspect is not taken into account.

### No major changes in temporal and spatial distribution of BVDV-1 genotypes in France

Statistical analysis suggests that there have been no clear changes in relative frequencies of genotypes 1e and 1b since the 1990s in France. This may indicate that the rate of introduction of BVDV-1 into France might not be high enough to replace established genotypes, or that such replacement through migration only occurred before the first molecular characterisation of circulating genotypes in France [13]. Furthermore, we showed that the relative frequencies of both main genotypes did not present major disparities, especially for the main cattle-producing regions. It could be the result of the great potential for virus dispersal through the dense cattle trade network. In addition, the presumably low effective migration rate could limit the introduction of other genotypes and slow the potential changes of genotype frequencies in the metapopulation of cattle herds.

France is currently implementing an eradication plan, which will presumably have an impact on the distribution of BVDV-1 genetic diversity. The plan should increase the removal of PI animals and therefore the rate of viral population extinction in subpopulations. Genetic diversity loss would then be expected, through an increase of genetic drift. The probability of losing a genotype would be directly linked to its frequency: the rarest genotypes would have the highest probability of being eliminated, while highly prevalent genotypes would take longer to be eradicated. This could explain why the main genotype tend to become even more predominant in the context of an eradication plan as seen in Switzerland [49] and, more strikingly, in Germany [48]. However, this effect is not yet visible in France, probably because of the gradual introduction of control measures and the latency in the implementation of the eradication plan, as well as the slow temporal dynamics of BVDV-1.

### Region-specific local spatial clustering in France

The spatial structuring of BVDV-1 genetic diversity is overall weak. Low positive Mantel correlations between genetic and geographic distances are significant only at short-distance classes for Normandie and Bretagne region (Figure 4D). This is consistent with what was observed previously: no correlation was reported in Switzerland [70], only at farm level in Austria [71] and there was evidence for local spread in nearby farms in England and Wales [72]. These studies have suggested the importance of cattle movements in the absence of geographical structuring. More generally, trade movements seem to be a driver of BVDV lineages distributions [58]. However, trade movements data could not be obtained for this study. Differences among the three French regions could further be explained by several factors: regional husbandry practices, location, the sampling scheme, and applied control measures. Western France (Normandie and Bretagne regions) has historically had very different productions systems and cattle density (mostly dairy and high cattle density) to those in AuRA (relatively balanced between dairy and beef and moderate cattle density) [73, 74]. These two factors have been shown to be risk factors [75–77] and could explain differences in spatial structuring between western France and AuRA. However, our sampling scheme was not suitable for specifically investigating the effect of production type (milk or beef) on genetic diversity, as dairy areas were over-represented in the data. Furthermore, the central geographical location of AuRA region in France could also explain the low spatial structuring in this region due to the potential border effect, involving cattle exchange with surrounding departments. As coastal regions, this effect is less expected for Bretagne and Normandie regions. The higher positive correlations observed in Normandie could be partly explained by the sampling scheme, as samples from the same herd were more likely to be included in this dataset than in Bretagne and AuRA datasets. Indeed, it has been shown that viruses sampled in the same herd for the same outbreak are almost genetically identical [63], which could increase correlation signal for short-distance classes. Nevertheless, our results suggest that, in Normandie region during the studied period (2016—2017), BVDV-1 genotypes could persist locally through transmission among neighbouring herds with few long-distance dispersal events. The positive short-distance correlation observed in Bretagne could be linked to compliance with control measures since 1998 in Bretagne [78], which could limit the risk of virus introduction when cattle are purchased from another herd and reinforce the importance of contacts with neighbours. This is consistent with the work by Qi et al. [76] which highlights the importance of neighbourhood contacts in virus diffusion in a modelling study calibrated on Bretagne data.

## Conclusion

This study presents an in-depth spatio-temporal analysis of BVDV-1 genetic diversity in France. It reveals distinct international patterns between the two main BVDV-1 circulating genotypes. Yet, we did not identify major changes in the proportions of these genotypes in France, either in space or over time. This study also shows that barcoding data generation, such as 5’UTR sequences, can be efficiently implemented to genetically monitor variants of an enzootic pathogen. However, the level of phylogenetic resolution associated with 5'UTR sequence data precludes advanced spatio-temporal analyses required to unravel BVDV-1 complex evolutionary and epidemiological dynamics. Genome-based studies would enable to explore the dynamics of the virus in greater details at different spatial and temporal scales, and make a better contribution to BVD disease management.

## Supplementary Information

**Additional file 1. Sample information.**

**Additional file 2. RNA extraction and BVDV 5’UTR sequencing details using Sanger protocol for datasets 1, 2 and 3.**

**Additional file 3. PCR conditions for (A) QIAGEN OneStep RT-PCR kit, (B) Q5 enzyme (New England Biolabs) and (C) Kapa Hifi enzyme in Nextera™ XT DNA Index Kit.**

**Additional file 4. Reference sequences used to assign a genotype.**

**Additional file 5. Validation of the 5’UTR high-throughput sequencing protocol.** (A) Relationship between the number of reads mapped and viral load (cycle threshold – Ct) per sample. Dots are coloured according to the sequencing run. (B) Relationship between percentage of reads mapped and viral loads (Cts) per sample. A polynomial regression was fitted for each run and the resulting R^2^ are displayed.

**Additional file 6. Genetic diversity of BVDV-1 in France.** Maximum likelihood phylogenetic tree was inferred from 1316 French isolates and international reference strains. Tips coloured in yellow and blue denote previously published French and reference sequences, respectively. Asterisks indicate samples that are identical or almost identical to vaccine strains. The tree was midpoint rooted. Solid bars at right correspond to BVDV-1 genotype delimitations based on reference strains. Statistical support for nodes were assessed using an ultrafast bootstrap (UFBoot, 1000 replicates) and a Shimodaira–Hasegawa approximate likelihood-ratio test (SH-aLRT, 1000 replicates). Diamonds at internal nodes denote well-supported clades with UFboot ≥ 95% and SH-aLRT >= 80% as suggested by IQ-TREE documentation.

**Additional file 7. Genotype and spatio-temporal distribution of publicly available BVDV-1 sequences.** (A) Location of publicly available sequences assigned to the main BVDV-1 genotypes circulating in France (1e, 1b, 1d, 1a and 1l). Each panel represent a genotype. Bar charts are coloured according to the continent of origin. The number of sequences per location is log-transformed. (B) Year of collection of publicly available sequences assigned to the main BVDV-1 genotypes circulating in France. Each panel represent a genotype. Bar charts are coloured according to the continent of origin. The number of sequences per year are log-transformed.

**Additional file 8. Phylogenetic relationships between French and international sequences of BVDV-1 genotype 1d.** Maximum likelihood phylogenetic tree inferred from French and international sequences of genotype 1d. Tips are coloured according to the sample continent or country of origin and their sizes represent the number of closely related publicly available sequences (99.5% identity) from the same location. European countries with less than ten sequences were grouped under the location “Europe” in the phylogeny.

**Additional file 9. Regional disparities in BVDV-1 genotype proportions.** Result of Fisher exact test (FET) post-hoc pairwise comparisons of genotype proportions (1e, 1b and other genotypes) between locations. (A) Tests were performed on all regions with a least 30 sampled sequences. AuRa and BCF codes correspond to Auvergne-Rhône-Alpes and Bourgogne-Franche-Comté regions, respectively. (B) Tests were performed on all Bourgogne-France-Comté departments with at least ten sampled sequences. Number in brackets correspond to department code.

**Additional file 10. Spatial distribution and local structure of BVDV-1 genotypes in two focused French regions with reduced temporal amplitude.** Proportions of BVDV-1 genotypes at local scale in (A) Bretagne, (B) and Auvergne-Rhône-Alpes. Dataset are truncated to include only two years of sampling for each region. Pie charts denote genotype proportions per farm postal code and sizes are proportional to the number of samples. A small amount of random variation was added to each pie chart location to ensure anonymity. Grey lines outline department borders. (C) Mantel correlograms measuring correlations between phylogenetic and geographical distances for the three focused regions. Each dot is positioned in the middle of a distance class. Statistically significant correlations are indicated by a square.

## Data Availability

All 5’UTR sequences from this study are available on NCBI under the accession numbers: PP513311 to PP514335. Farm postal codes for Bretagne and AuRA regions are available on request from the authors and after consultation with the GDS.
