# Supplementary material for: Spatio-temporal distribution and international context of bovine viral diarrhoea virus genetic diversity in France
Source: Vet Res. 2024 Oct 3;55:129. doi: 10.1186/s13567-024-01377-9 (PMC11451180; doi:10.1186/s13567-024-01377-9)
Supplement: Supplementary file 2 — Additional file 2. RNA extraction and BVDV 5’UTR sequencing details using Sanger protocol for datasets 1, 2 and 3. [file 13567_2024_1377_MOESM2_ESM.docx]

##### BVDV 5’UTR sequencing

**Dataset 1.** RNA extractions were performed depending on sample nature. Organ samples were ground and filtered on a 0.45µm Millex membrane (Merck). Viral RNA was extracted from lysed whole blood, filtrates and tank milks with the NucleoMag 96 kit (BIO-X) on the KingFisher automated system (ThermoFisher) according to the supplier's instructions. Ear notches were lysed with the Bio-X Adiapure TLB kit (BioX diagnostics) and then amplified directly. A specific fragment of 5’UTR region of BVDV genome was amplified by PCR using primers 324F and 326R as previously described in [16]. Sanger sequencing was outsourced to Biofida (France).

**Dataset 2.** RNA extractions were performed using MagMax Core nucleic acid purification kit (ThermoFisher) on a KingFisher Duo Prime automated system (ThermoFisher). Reverse transcription and 5’UTR PCR amplification were performed using One step RT PCR kit (Qiagen Inc.). The RT-PCR mix consisted of 324F and 326R primers used at a final concentration of 0.3 µM, dNTPs used at a final concentration 0.3 µM, 2µL QIAGEN OneStep RT-PCR Enzyme Mix and 10uL QIAGEN OneStep RT-PCR Buffer, 5x. PCR amplification was performed as described in table 2A on a thermocycler CFX96 (Bio-rad). Sanger sequencing was performed in ANSES laboratory of Ploufragan (France).

**Dataset 3.** Ear notches nucleic acids were extracted using the MagVet™ Universal Isolation Kit (Innovative Diagnostics) for samples from September 2019 up to September 2022 and ID Gene Mag Fast Extraction Kit (Innovative Diagnostics) after September 2022. Serum RNA extraction was performed using NucleoSpin RNA Virus Mini kit for viral RNA (Macherey Nagel). For 96 samples of dataset 3, including 19 serums, 4 µL of nucleic acids were denatured at 65 °C for 5 min with 1 µL of primers 326R (10 uM). Denatured nucleic acids were reverse-transcribed using ProtoScript II Reverse Transcriptase (New England Biolabs) as described by the manufacturer. Enzyme was inactived at 80 °C for 5 min. PCR amplification was performed with Q5 polymerase enzyme (New England Biolabs) using temperature profile detailed in Table 2B. Results were visualized on a 1% agarose gel. Amplicons of the expected size were sent to Eurofins for Sanger sequencing. The remaining 282 samples were sequenced using the high-throughput sequencing protocol developed in the study.

**Dataset 4.** Extraction was carried out using BioExtract® SuperBall® kit (Biosellal). Amplification was performed with BioT kit® BVDV/BDV Universal (Biosellal). All samples were sequenced using the high-throughput sequencing protocol developed in the study.
