# Supplementary figures and images for: Spatio-temporal distribution and international context of bovine viral diarrhoea virus genetic diversity in France
Source: Vet Res. 2024 Oct 3;55:129. doi: 10.1186/s13567-024-01377-9 (PMC11451180; doi:10.1186/s13567-024-01377-9)

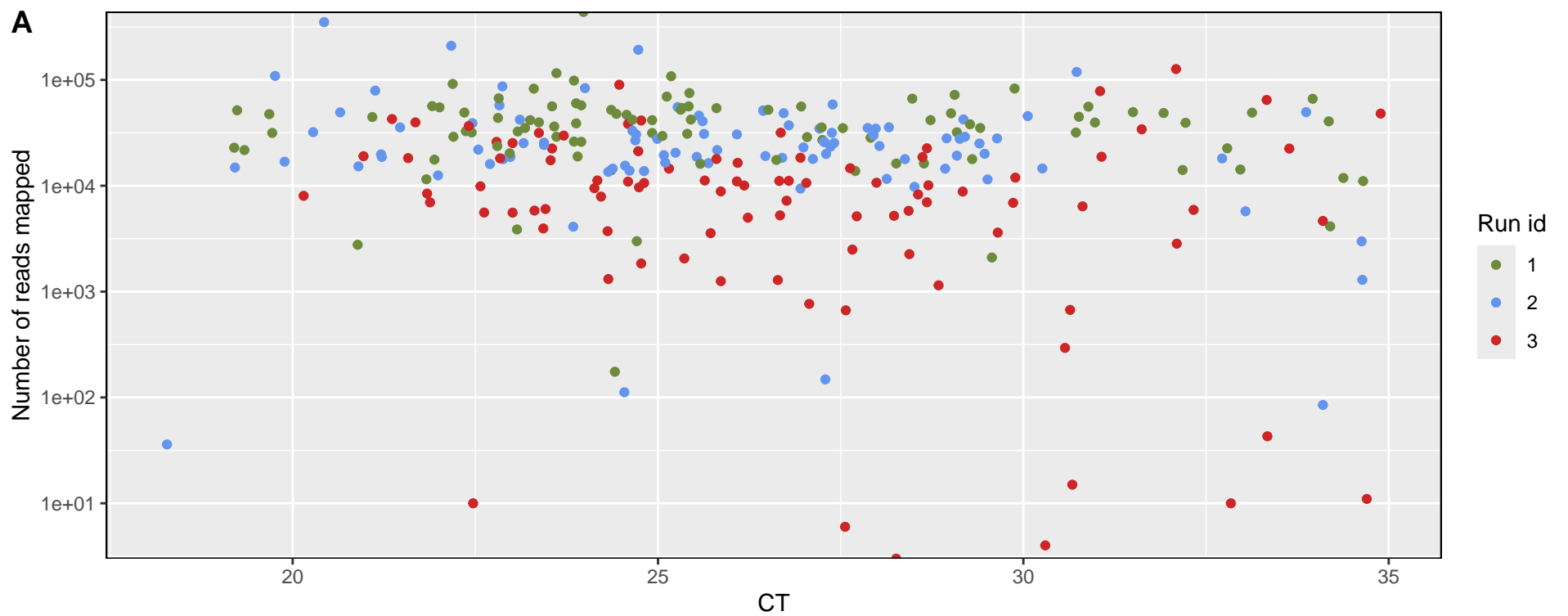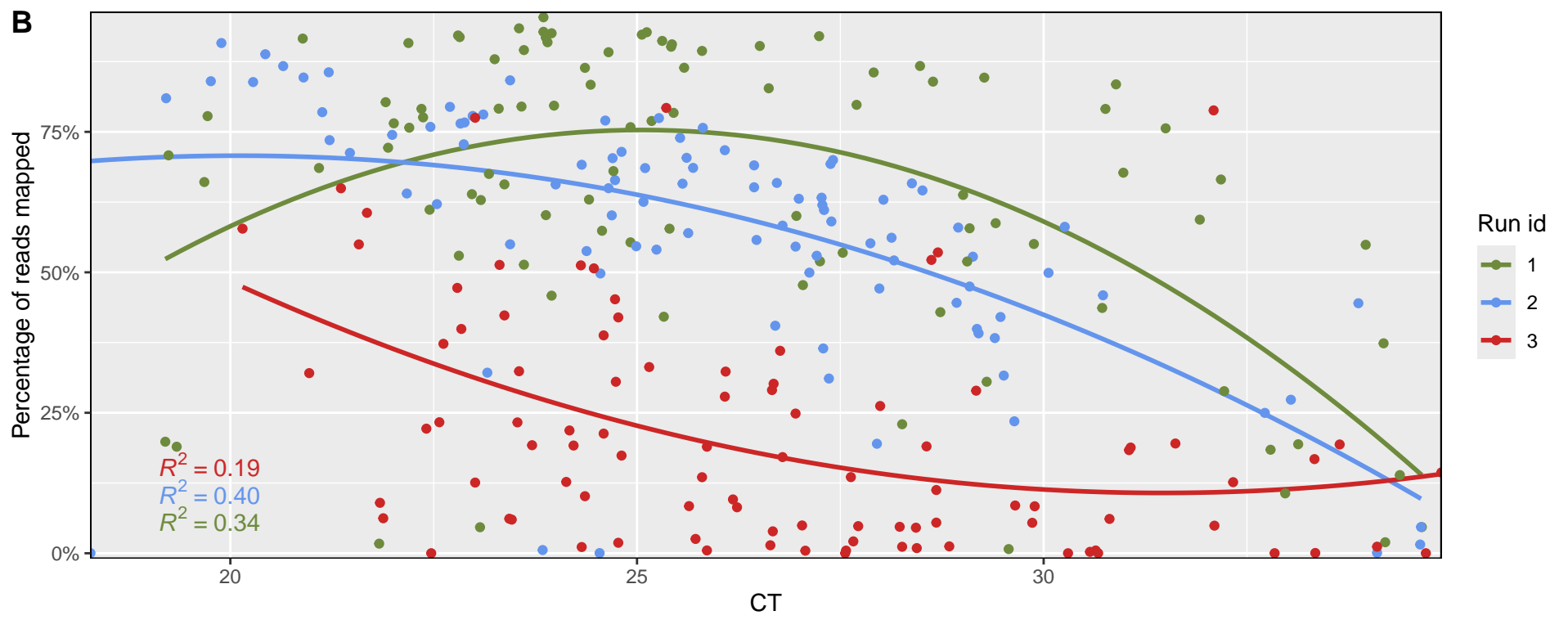

Supplement: Supplementary file 5 — Additional file 5. Validation of the 5’UTR high-throughput sequencing protocol. (A) Relationship between the number of reads mapped and viral load (cycle threshold – Ct) per sample. Dots are coloured according to the sequencing run. (B) Relationship between percentage of reads mapped and viral loads (Cts) per sample. A polynomial regression was fitted for each run and the resulting R2 are displayed. [file 13567_2024_1377_MOESM5_ESM.pdf]

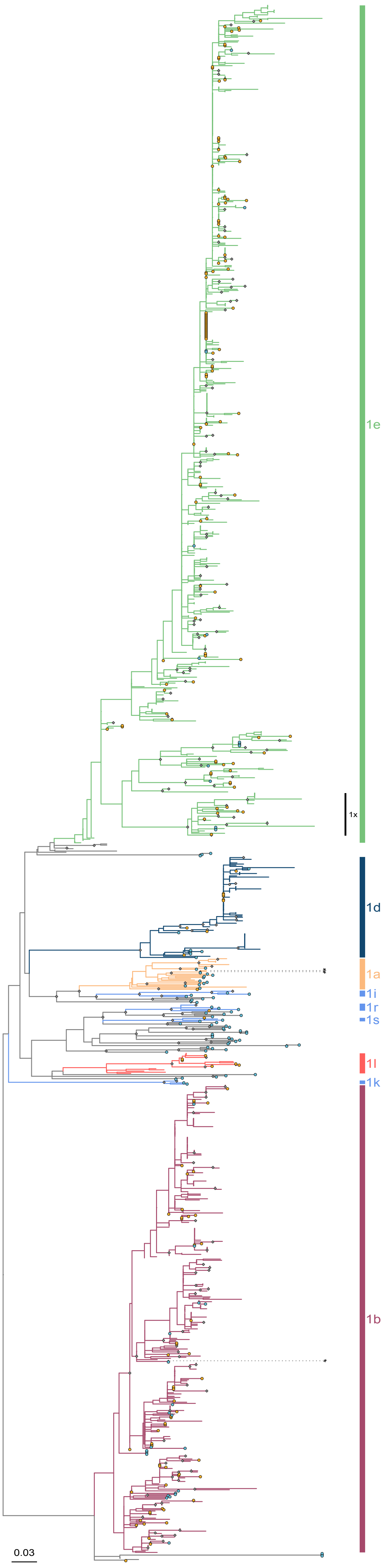

Supplement: Supplementary file 6 — Additional file 6. Genetic diversity of BVDV-1 in France. Maximum likelihood phylogenetic tree was inferred from 1316 French isolates and international reference strains. Tips coloured in yellow and blue denote previously published French and reference sequences, respectively. Asterisks indicate samples that are identical or almost identical to vaccine strains. The tree was midpoint rooted. Solid bars at right correspond to BVDV-1 genotype delimitations based on reference strains. Statistical support for nodes were assessed using an ultrafast bootstrap (UFBoot, 1000 replicates) and a Shimodaira–Hasegawa approximate likelihood-ratio test (SH-aLRT, 1000 replicates). Diamonds at internal nodes denote well-supported clades with UFboot ≥ 95% and SH-aLRT >= 80% as suggested by IQ-TREE documentation. [file 13567_2024_1377_MOESM6_ESM.pdf]

A

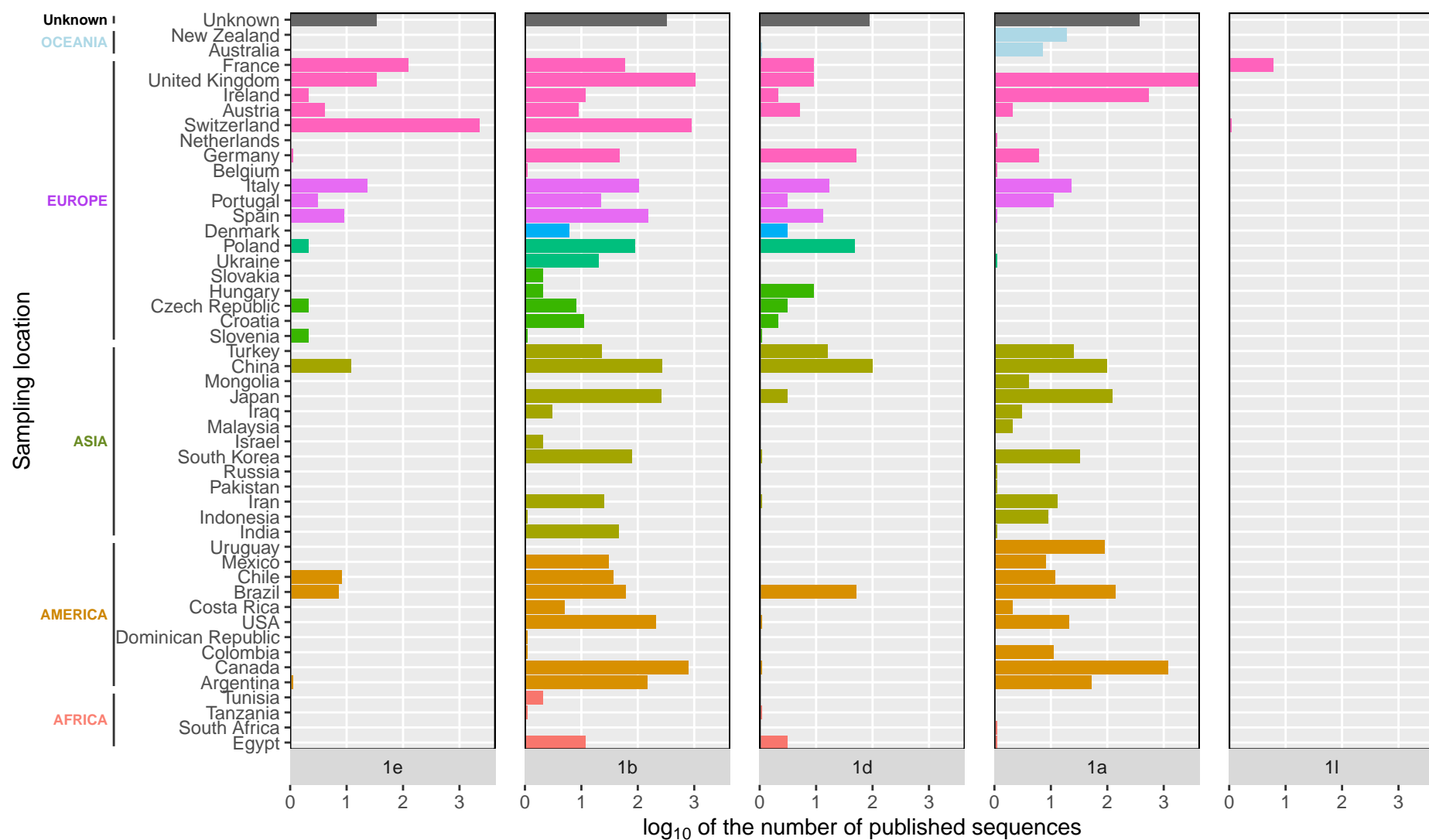

B

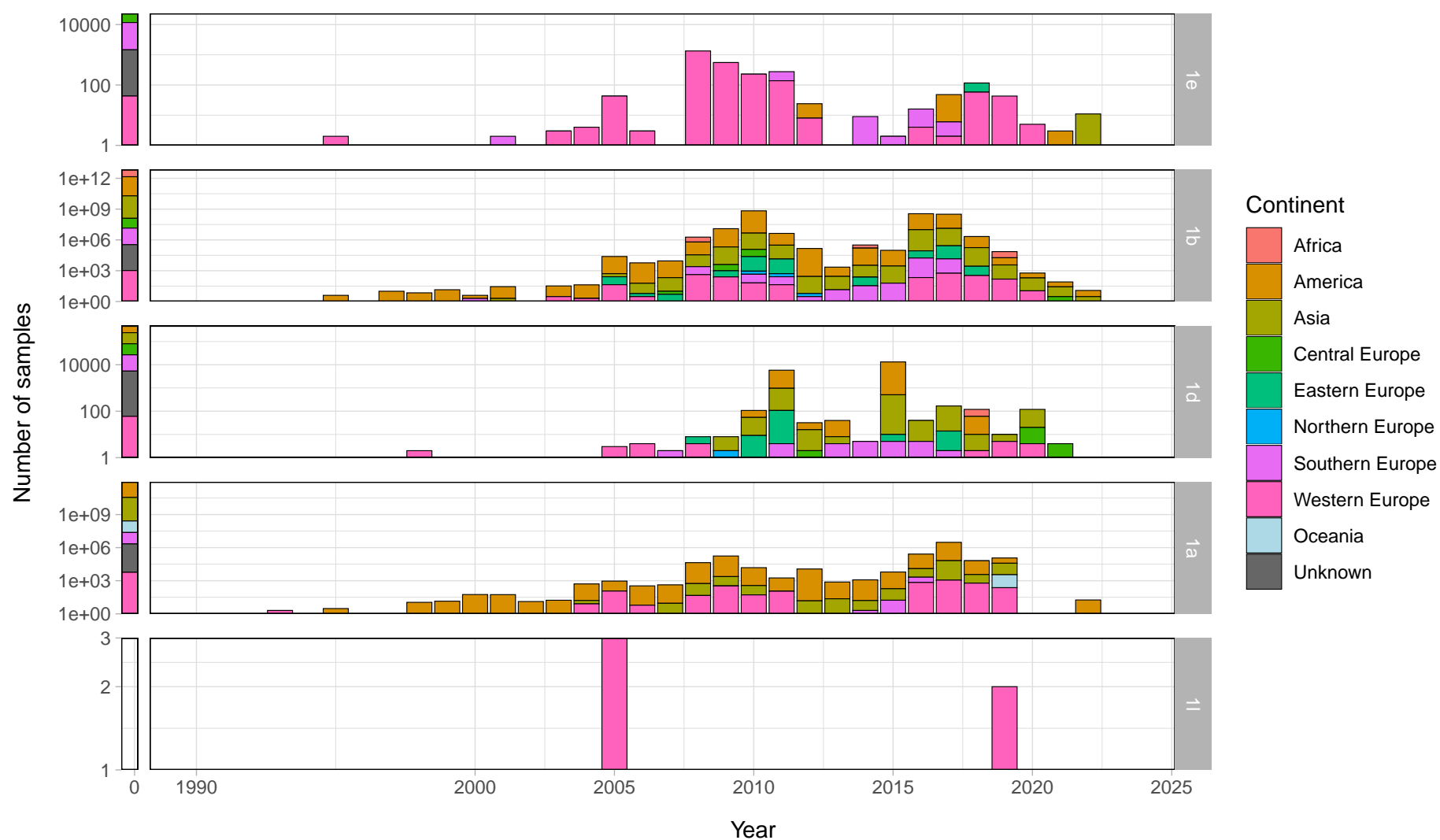

Supplement: Supplementary file 7 — Additional file 7. Genotype and spatio-temporal distribution of publicly available BVDV-1 sequences. (A) Location of publicly available sequences assigned to the main BVDV-1 genotypes circulating in France (1e, 1b, 1d, 1a and 1l). Each panel represent a genotype. Bar charts are coloured according to the continent of origin. The number of sequences per location is log-transformed. (B) Year of collection of publicly available sequences assigned to the main BVDV-1 genotypes circulating in France. Each panel represent a genotype. Bar charts are coloured according to the continent of origin. The number of sequences per year are log-transformed. [file 13567_2024_1377_MOESM7_ESM.pdf]

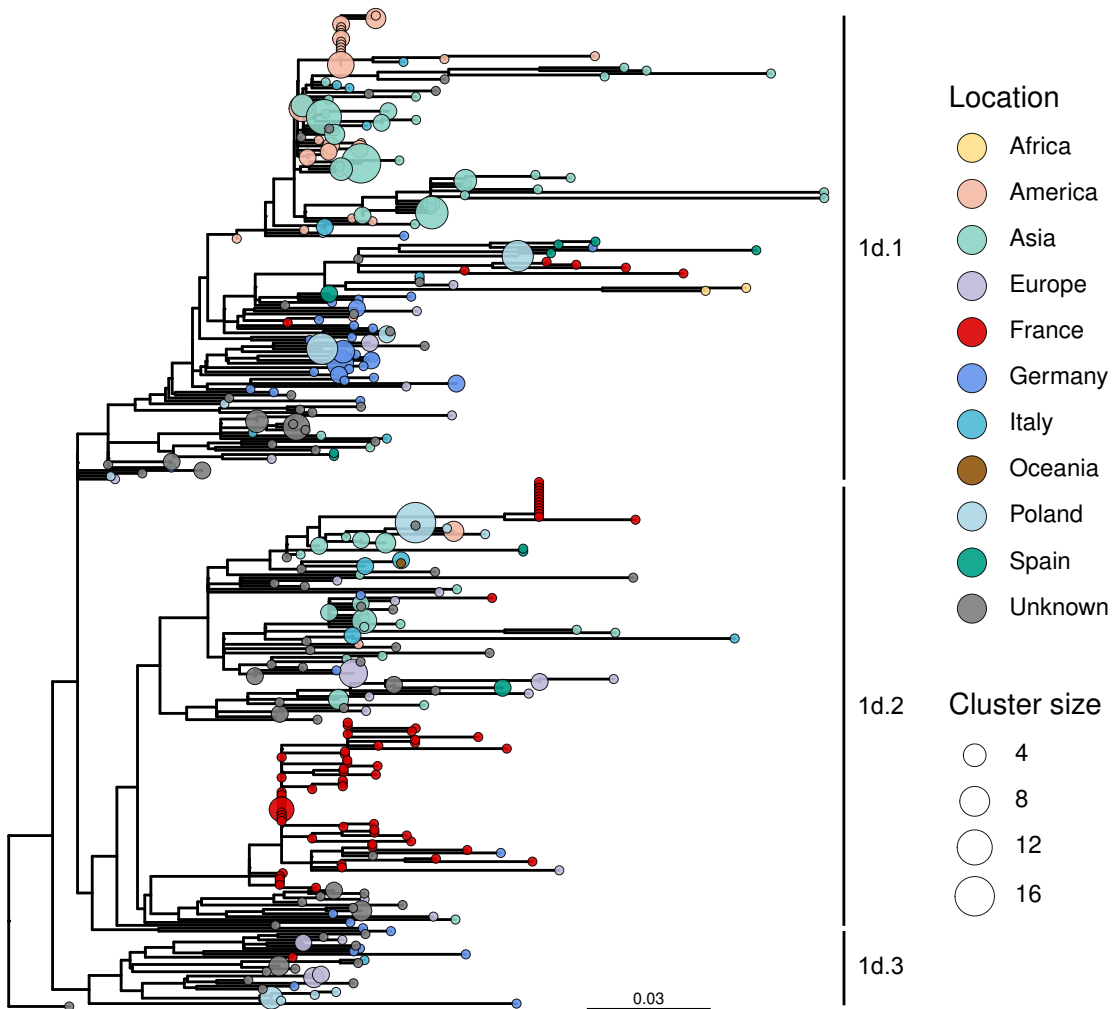

Supplement: Supplementary file 8 — Additional file 8. Phylogenetic relationships between French and international sequences of BVDV-1 genotype 1d. Maximum likelihood phylogenetic tree inferred from French and international sequences of genotype 1d. Tips are coloured according to the sample continent or country of origin and their sizes represent the number of closely related publicly available sequences (99.5% identity) from the same location. European countries with less than ten sequences were grouped under the location “Europe” in the phylogeny. [file 13567_2024_1377_MOESM8_ESM.pdf]

**A**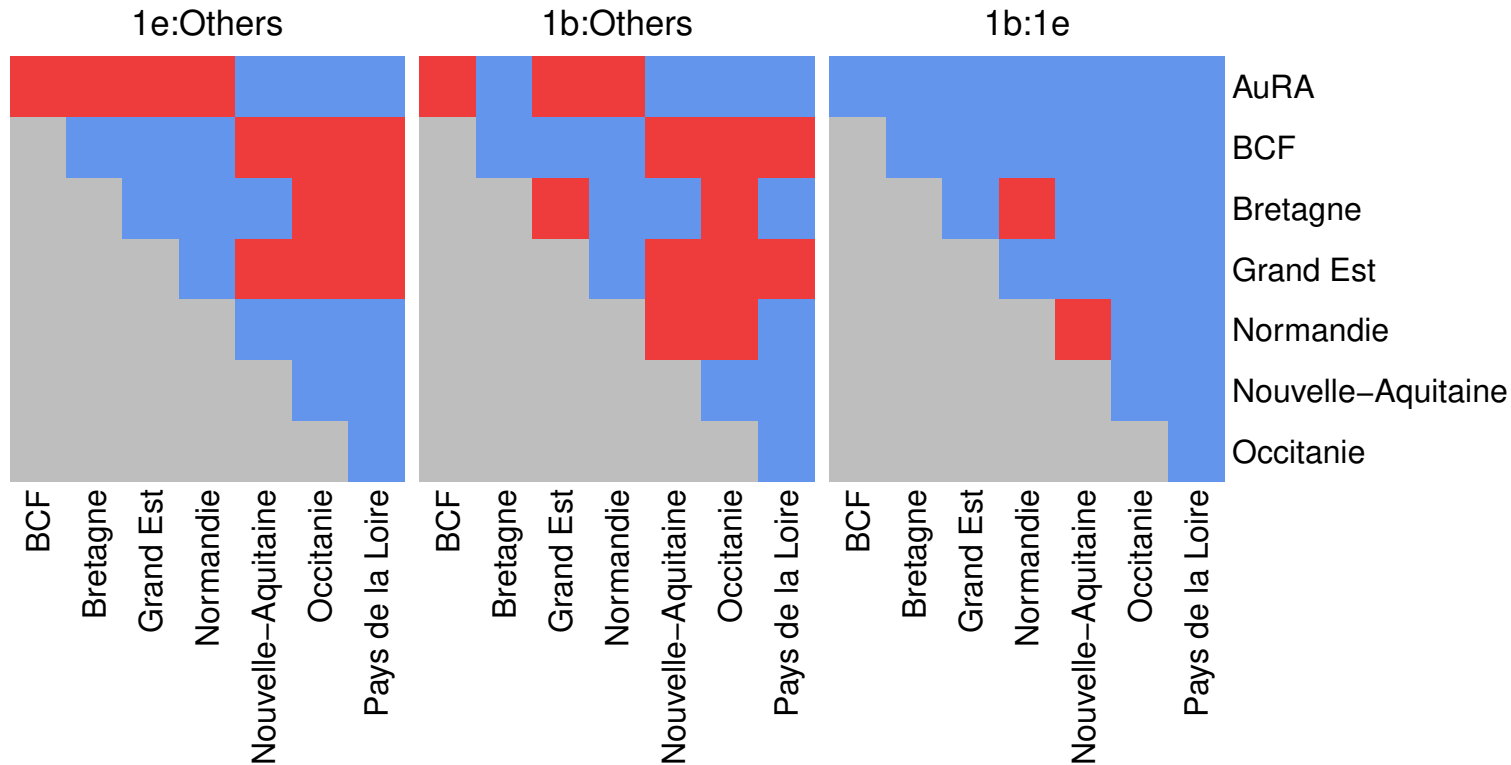**B**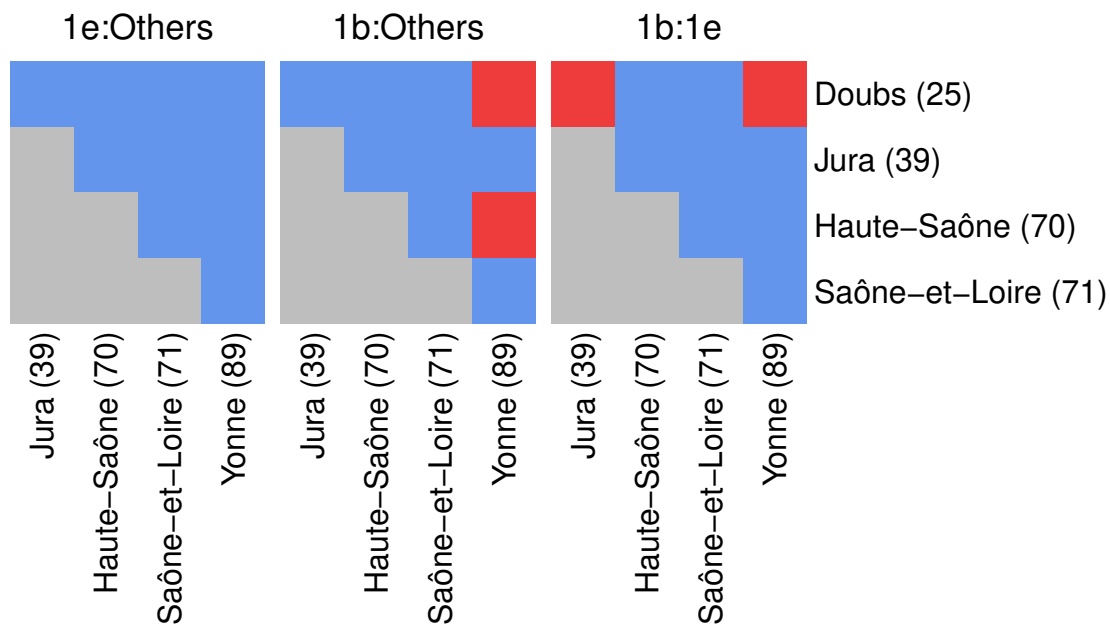**Significance**

Significant ( $p\text{-val} < 0.05$ )  
Not significant

Supplement: Supplementary file 9 — Additional file 9. Regional disparities in BVDV-1 genotype proportions. Result of Fisher exact test (FET) post-hoc pairwise comparisons of genotype proportions (1e, 1b and other genotypes) between locations. (A) Tests were performed on all regions with a least 30 sampled sequences. AuRa and BCF codes correspond to Auvergne-Rhône-Alpes and Bourgogne-Franche-Comté regions, respectively. (B) Tests were performed on all Bourgogne-France-Comté departments with at least ten sampled sequences. Number in brackets correspond to department code. [file 13567_2024_1377_MOESM9_ESM.pdf]

**A**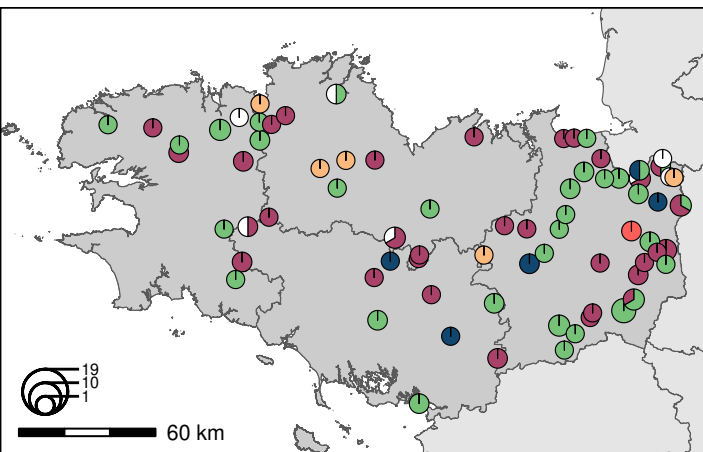**B**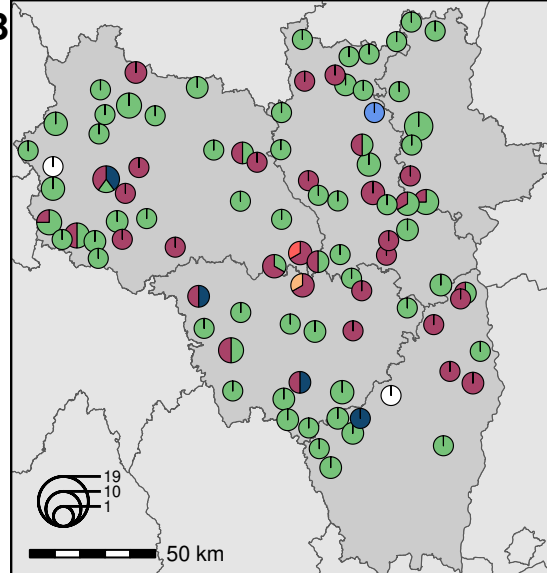**C**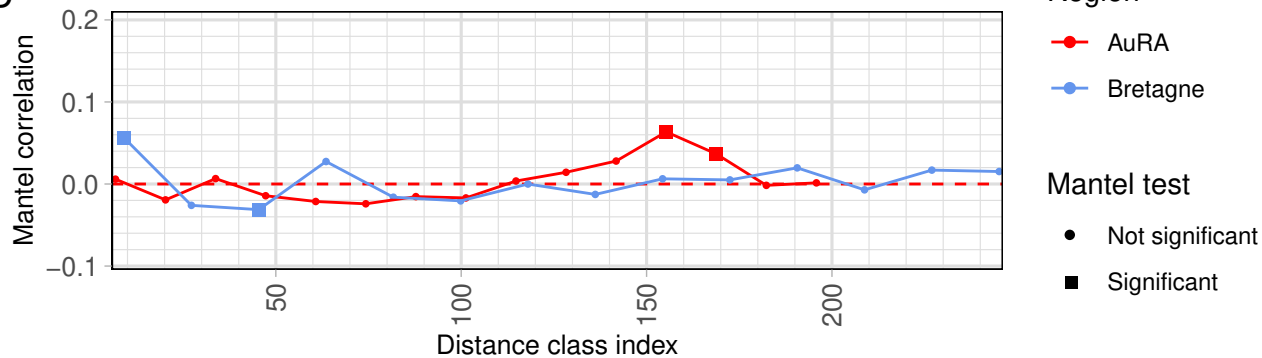

Supplement: Supplementary file 10 — Additional file 10. Spatial distribution and local structure of BVDV-1 genotypes in two focused French regions with reduced temporal amplitude. Proportions of BVDV-1 genotypes at local scale in (A) Bretagne, (B) and Auvergne-Rhône-Alpes. Dataset are truncated to include only two years of sampling for each region. Pie charts denote genotype proportions per farm postal code and sizes are proportional to the number of samples. A small amount of random variation was added to each pie chart location to ensure anonymity. Grey lines outline department borders. (C) Mantel correlograms measuring correlations between phylogenetic and geographical distances for the three focused regions. Each dot is positioned in the middle of a distance class. Statistically significant correlations are indicated by a square. [file 13567_2024_1377_MOESM10_ESM.pdf]
